# Robust memory of face moral values is encoded in the human caudate tail: a simultaneous EEG-fMRI study

**DOI:** 10.1038/s41598-024-63085-w

**Published:** 2024-06-01

**Authors:** Ali Ataei, Arash Amini, Ali Ghazizadeh

**Affiliations:** 1 EE Department, Sharif University of Technology, Azadi Avenue, Tehran, 1458889694 Iran; 2 Sharif Brain Center, Sharif University of Technology, Tehran, Iran; 3 School of Cognitive Sciences, Institute for Research in Fundamental Sciences, Tehran, Iran

**Keywords:** Neuroscience, Cognitive neuroscience, Learning and memory

## Abstract

Moral judgements about people based on their actions is a key component that guides social decision making. It is currently unknown how positive or negative moral judgments associated with a person’s face are processed and stored in the brain for a long time. Here, we investigate the long-term memory of moral values associated with human faces using simultaneous EEG-fMRI data acquisition. Results show that only a few exposures to morally charged stories of people are enough to form long-term memories a day later for a relatively large number of new faces. Event related potentials (ERPs) showed a significant differentiation of remembered good vs bad faces over centerofrontal electrode sites (value ERP). EEG-informed fMRI analysis revealed a subcortical cluster centered on the left caudate tail (CDt) as a correlate of the face value ERP. Importantly neither this analysis nor a conventional whole-brain analysis revealed any significant coding of face values in cortical areas, in particular the fusiform face area (FFA). Conversely an fMRI-informed EEG source localization using accurate subject-specific EEG head models also revealed activation in the left caudate tail. Nevertheless, the detected caudate tail region was found to be functionally connected to the FFA, suggesting FFA to be the source of face-specific information to CDt. A further psycho-physiological interaction analysis also revealed task-dependent coupling between CDt and dorsomedial prefrontal cortex (dmPFC), a region previously identified as retaining emotional working memories. These results identify CDt as a main site for encoding the long-term value memories of faces in humans suggesting that moral value of faces activates the same subcortical basal ganglia circuitry involved in processing reward value memory for objects in primates.

## Introduction

Our past experiences with people whether good or bad affect our future interactions with them and guide our social decision making. In particular, moral judgement about people based on their actions is a key component in our evaluation of an individual^1–4^. It is currently unknown how positive or negative moral judgments associated with a person’s face are processed and stored in the brain as a long-term memory.

Human brain has specialized face processing areas in the temporal cortex including the occipital face area (OFA), superior temporal sulcus (STS) and the fusiform face area (FFA)^5,6^. Several studies have examined the coding of various aspects of faces based on their intrinsic visual features such as emotional expressions^7,8^, attractiveness^9–13^ trustworthiness^14,15^ or social value^16,17^ in the human brain with somewhat divergent results. A review and meta-analysis of these findings suggests consistent activations for negative evaluations in the amygdala, and for positive evaluations in the medial orbitofrontal cortex (mOFC), anterior cingulate cortex (ACC), caudate nucleus and the nucleus accumbens (NAcc)^18^.

The value circuitry for objects in general is extensively studied in both humans and non-human primates with key cortical and subcortical areas such as orbitofrontal cortex (OFC), insula, ACC, basal ganglia, amygdala and midbrain dopaminergic areas being activated during object reward association tasks^19–24^. Value memory is also shown to activate a temporal-prefrontal circuitry along with its functionally connected subcortical areas, in particular, the caudate nucleus, amygdala and claustrum^25–30^. Interestingly, these studies have illuminated some different aspects of the short and long-term value memories. Specifically, it is shown that while the head of caudate nucleus exclusively represents the flexible and short-term memory of object values, the tail of caudate nucleus exclusively represents the stable and long-term memory of object values in monkeys^24^. Replication of the same experimental task on humans using fMRI^28,31^ has revealed activities in the ventral striatum, caudate body and hypothalamus. While there are some recent studies that have looked at short-term effects of value assignment to faces using EEG or fMRI^32–39^, it is not known how long-term memory of associated value with faces engages this circuitry and whether it can change even the primary face processing in areas such as FFA.

To address this question, we randomly assigned positive or negative values based on morally charged biographical stories to novel faces and then examined the brain activations to these faces a day later using a simultaneous EEG-fMRI paradigm which involved a binary choice for face values. The value ERP showed a significant differentiation of the correctly identified good and bad faces over center-frontal electrodes, peaking at about 600 ms post-stimulus and lasting until almost the end of face presentation. This ERP is shown to originate from the left caudate tail, based on results from both EEG-informed fMRI analysis and fMRI-informed EEG source localization. Interestingly, while none of the cortical face processing areas were found to encode the moral values of faces, some were found to be functionally connected to the detected value-coding caudate tail region.

## Results

**Figure 1:**
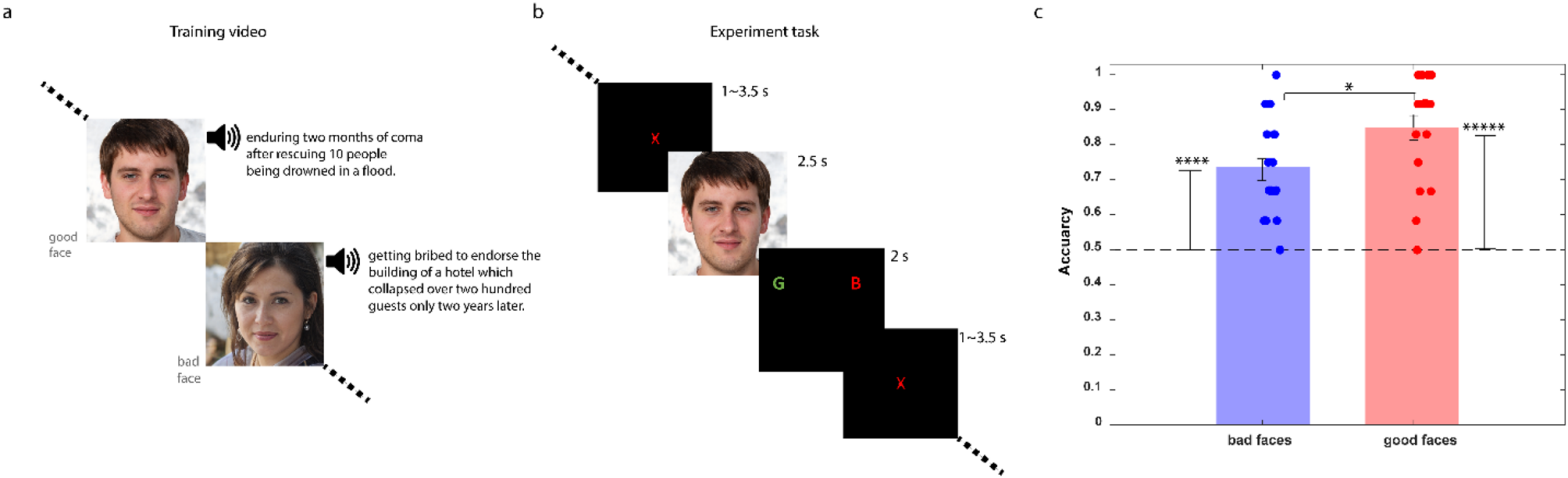
Training procedure, memory task and behavioral results.(**a**) Value training video: In this video, 24 novel faces were associated with short biographical stories, each conveying a positive or negative moral value about the presented face (good or bad faces, see Suppl. Table 1 for the list of morally-charged stories). (**b**) Memory task: faces seen the day before were shown in a random sequence for each subject. Each face was shown for 2.5 s. Then, a black screen was shown with two letters of “G” and “B” (referring to “good” and “bad”, respectively) for 2 s during which, the subject had to indicate his/her response by pressing the appropriate button. The sides of letters “G” and “B” were randomly flipped in each trial. Then, an inter-stimulus-interval (a black screen with a red fixation cross at center) followed for a random time period between 1 to 3.5 s. (**c**) Behavioral result: The performance of subjects in judging face values, along with the chance level (= 50%, dashed line) is shown. Individual subject data points are shown. Error bars indicate standard errors.

To create value memory for faces, 24 faces were randomly associated with brief unique biographies with either a positive (good faces) or a negative (bad faces) moral value (see Suppl. table 1 for a list of all stories). In the value training session (Fig. 1a), subjects viewed each face while listening to the associated short story. One day later, the face value memory of subjects were examined using a simultaneous EEG-fMRI paradigm. During the experimental task, each face was portrayed for 2.5 s (Fig. 1b). Then the face disappeared and the subject was asked about the moral value of the recently observed face. The subject had to indicate his/her response by pressing a response button using the corresponding hand within 2 s. The assignment of a given face to good or bad category was counterbalanced among subjects to ensure that value judgements were driven by the associated biographies and not confounded by physical features of faces.

The subjects’ accuracy in identifying face types was 78% on average, which is significantly higher than the chance level. The subjects’ accuracies for each specific category were also significantly high (Fig. 1c; *t*-test; t(19) = 7.33, p < 1e−6 & t(19) = 10.45, p < 1e−8, for bad and good faces respectively). This suggests that a brief exposure to a large number of new people (24 new faces) and their moral stories creates a lasting memory that is accessible a day later. Nevertheless, the performance for the good faces was significantly higher than that for bad ones by about 12% (Fig. 1c, paired *t*-test; t(19) = 3.3, p = 0.004). To ensure that there was no systematic bias for some faces to be remembered as good or bad, we took advantage of the fact that a given face value was counterbalanced among subjects and looked at the percentage of times a certain face was chosen as good or bad across subjects (including additional subjects outside the EEG-fMRI experiment, n = 34). Results showed that among the 24 faces used, only 2 showed significant bias to be chosen as good (Suppl. Table 3). We conducted our subsequent analysis both by inclusion and exclusion of these 2 faces, which did not change any of our main findings as will be discussed.

## Robust differentiation of remembered good and bad faces over center-frontal electrodes

**Figure 2:**
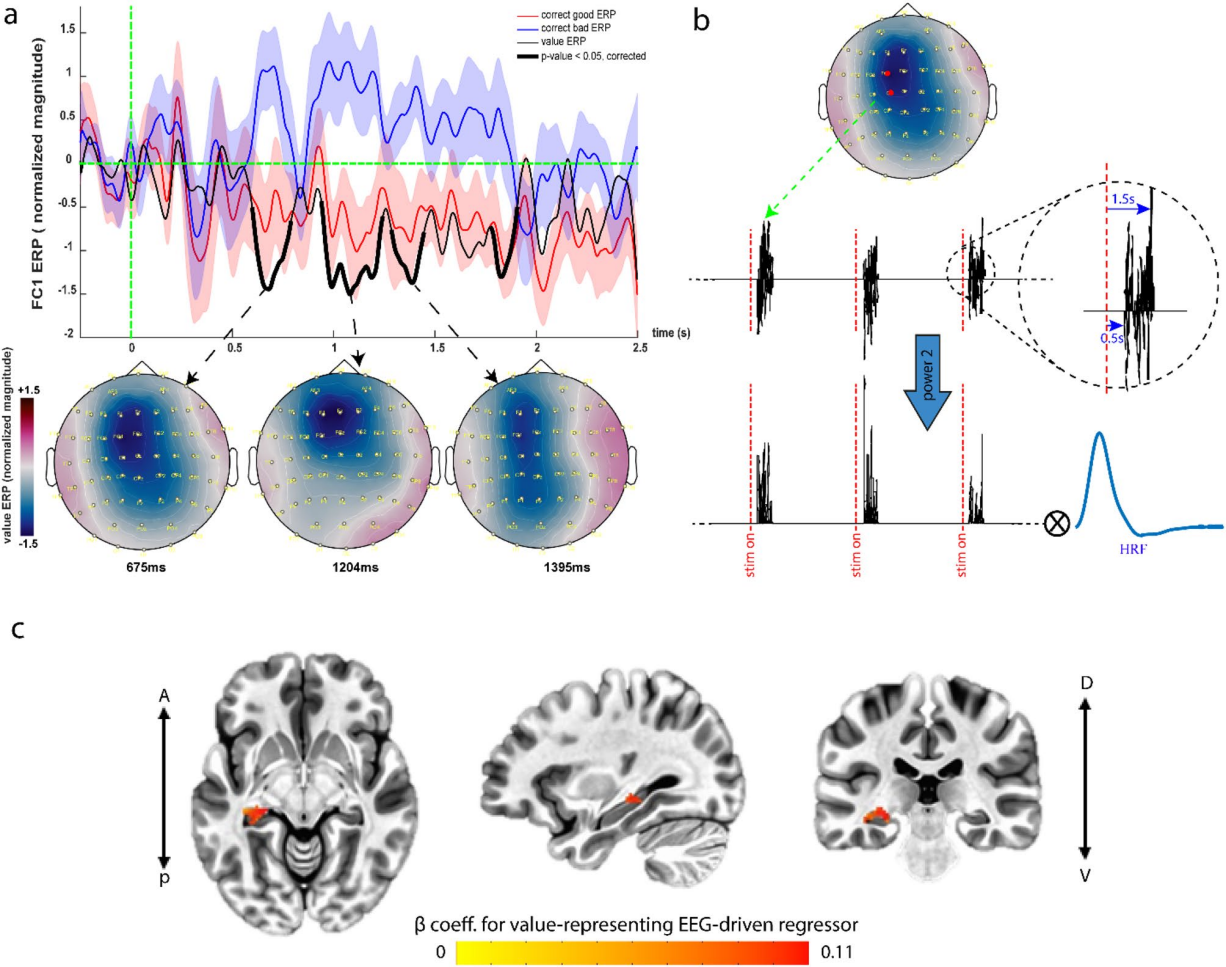
Value ERP and the EEG-informed fMRI analysis. (**a**) The group-average ERPs for the memorized good and bad faces and their difference (value ERP) in electrode FC1 along with the associated group-average scalp potential topographies at some sample time points. Parts of the value ERP that are significantly different from its baseline are shown with bolded black segments (**b**) The average of electric potentials across center-frontal electrodes over the significant interval (0.5–1.5 s post-stimulus) is considered as the EEG-driven value signal. This signal is then squared and consequently convolved with the canonical HRF to build our EEG-driven regressor. (**c**) EEG-informed fMRI analysis: the group-average of the beta coefficients for the EEG-driven regressor shows significant activation in a cluster centered on the left caudate tail.

To study the differential neuronal activity during remembered good and bad faces, event-related potentials (ERPs) for each category, separately were measured. In order to create a more precise group average, the EEG signals for each subject were normalized and transferred to the standard cap situated over the standard brain (see “group-average of ERPs” in methods for details). The ERPs for both good and bad faces in electrode P8, showed significant negativities around 230 ms post stimulus (lasting from about 190 ms to 250 ms, Suppl. Figure 2a, b; one-sided *t*-test against baseline; t(249) > 1.66, p-value < 0.05, FDR-corrected) consistent with previously reported N170 and early posterior negativity (EPN) components^36,38^. The normalized z-scored ERPs for the remembered good and bad faces, as well as their difference (the value ERP) showed a robust differentiation between good and bad faces over a large portion of the 2.5 s stimulus presentation on center-frontal channels such as C1 and FC1 (Fig. 2a; one-sided *t*-test against baseline; t(249) > 1.66, p-value ≤ 0.05, Bonferroni corrected over channels and time). This value dissociation is more strongly evident in the interval of 0.5 to 1.5 s post-stimulus (raw non-normalized ERPs showed a similar value differentiation with a similar time course, Suppl. Figure 2c).

## EEG-informed fMRI analysis reveals caudate tail as the origin of the value ERP

We next performed an EEG-informed fMRI analysis aiming to find the BOLD correlates of the value ERP found in the EEG signals, across whole brain voxels. Based on the time course of value ERP, we used the EEG signal averaged across C1 and FC1 electrodes in the interval from 0.5 to 1.5 s post-stimulus as our EEG-driven regressor in the fMRI GLM model (Fig. 2b, see methods). We have previously shown that the square (or power 2) of the EEG signals is the optimal regressor of the BOLD responses^40^. Thus, to localize the source(s) of EEG signals in the center-frontal electrodes, its square was used as the *trial by trial* correlate of face value signal in the brain (Fig. 2b).

Other regressors in the model included correct answer to good faces, “correct good”, correct answer to bad faces, “correct bad”, incorrect answer to good faces, “incorrect good” and incorrect answer to bad faces, “incorrect bad”. Moreover, to account for the effect of subject’s motor action and possible differences in response latency during button press, we also added two stick function trains convolved with standard hemodynamics at the times of responses with each hand. Finally, the average BOLD signal inside the ventricles was used as another nuisance regressor to ensure that responses in structures near the ventricles are not affected by such nonneural extraneous signals (GLM1, see materials & methods for details of the regressor design). As a control analysis, the right versus left hand contrast showed significant activation in the contralateral and deactivation in the ipsilateral motor cortices (Suppl. Figure 3a; t(15) > 2.94, p-value < 0.01, cluster-corrected). Moreover, activation of the ipsilateral and deactivation of the contralateral cerebellar cortices were also observable (Suppl. Figure 3b; t(15) > 2.94, p-value < 0.01, cluster-corrected), consistent with existing literature^41^.

The BOLD correlate of the EEG-driven regressor showed significant activation only in one subcortical cluster centered on the left caudate tail (CDt) (Fig. 2c; t(15) > 2.94, p-value < 0.01, cluster-corrected). Notably, the contrast of correct good vs. correct bad (the value contrast) in the presence of the EEG regressor did not reveal any additional activation in the brain suggesting that value contrast was well captured by the EEG power of center-frontal electrodes in each trial. This is expected since a correct model of statistical dependence among variables predicts that given the EEG signal originating from value contrast, BOLD should become independent of value contrast itself (Suppl. Figure 4a).

To ensure that CDt is indeed activated by the value contrast, we repeated our GLM analysis without an EEG-driven regressor (GLM2). As expected, in this case correct good vs. correct bad contrast showed significant activation in the left CDt (Suppl. Figure 4b; t(20) > 2.84, p-value < 0.01, cluster-corrected ). We note however that the extent of activation in this case was broader (including parts of the posterior hippocampus, Table 1) compared to the more localized CDt activation seen in the EEG-informed analysis which we believe attests to the advantage of simultaneous EEG-fMRI paradigm in finding more localized activations in fMRI. Importantly, we observed no other significant subcortical or cortical value differentiations in either GLM1 or GLM2, suggesting a special role for CDt in representing long-term memory of moral values of faces. Repeating GLM1 analysis by excluding the two faces that had a choice bias across subjects did not change our results confirming the activations found in the left caudate tail. (Suppl. Figure 5; t(15) > 2.94, p-value < 0.01, cluster-corrected).

**Table 1:** Activated clusters. Sizes, coordinates and cluster-correction thresholds for the activated clusters.

	Cluster size (voxels)	CM x	CM y	CM z	Peak x	Peak y	Peak z	Cluster threshold for p = 0.01 (voxels)
corr. good-corr. bad (GLM2):								66
L caudate tail	86	29.3	38.3	−1.2	32	38	−4	
EEG-driven regressor (GLM1):								54
L caudate tail	55	29.8	27.5	−6.1	28	26	−4	
EEG-driven regressor (GLM3):								56
L caudate tail	129	29.5	28.7	−8.8	22	26	−10	
correct-incorrect:								74
L angular gyrus	296	47.7	59.5	35.1	46	56	34	
L superior frontal gyrus	151	18.7	−27.8	52.2	18	−28	58	
L medial temporal gyrus	75	64	25.9	−7.3	62	26	−8	
right hand-left hand								80
R motor cortex	1393	−37.8	25	56.1	−52	16	56	
L cerebellum	655	17.5	52.7	−21	26	50	−22	
L lingual cortex	583	19.2	65	−8.9	16	74	−10	
R insula	447	−45.6	16.4	13.9	−48	14	16	
R cerebellum	328	−20.6	48.9	−22.5	−20	50	−22	
L motor cortex	318	36.9	23.3	57.5	36	22	44	
L cerebellum2	261	16.3	60.3	−47	16	58	−46	
R cingulate gyrus	254	−7.6	10.4	50	−10	26	48	
R posterior cingulate	242	−12.7	59.8	3.6	−4	74	8	
R Cuneus	215	−13.1	93.7	4	−16	94	0	
L Cuneus	200	19	86.3	23.9	12	86	28	
L Cuneus2	164	8	76.8	9.5	10	70	6	
R thalamus	149	−10.9	18.4	9.7	−10	20	12	
R superior temporal gyrus	117	−55.5	16.1	7.6	−50	18	10	
L Cuneus3	112	3	87.4	10.5	0	90	18	
R inferior frontal gyrus	84	−59.7	−7.3	17.9	−60	−12	22	

*CM* center of mass.

## fMRI-informed EEG source localization confirms left CDt as the origin of the value ERP and reveals its activation time course

**Figure 3:**
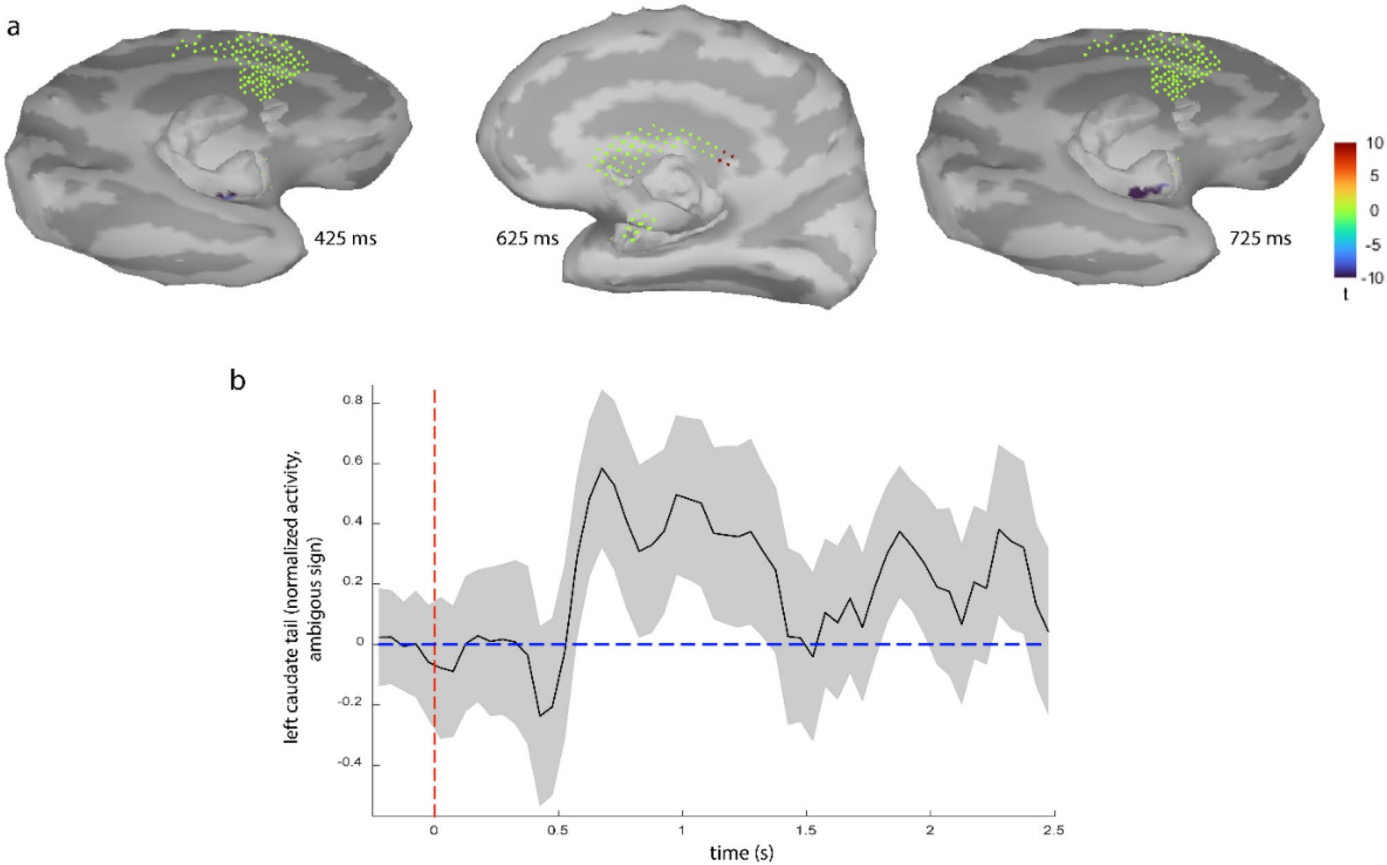
EEG sources of the value ERP. (**a**) The t-stat maps for the group average of the sources of value ERP, against their baseline activities. Note that subcortical regions with unknown dipole directions (caudate & amygdala) are shown as grid points along with the *opposite* cortical hemispheres. This map shows activation of the left caudate tail and deactivation of anterior hippocampus (note the ambiguity in the sign of dipole activities in caudate). (**b**) The group-average time course of the activity in the caudate tail (normalized magnitude) along with standard error margins indicating group diversity around the mean.

The spatial resolution of EEG is low and localizing its sources is highly sensitive to the implemented head model and the method used to solve the ill-posed inverse problem^42,43^. However, in our setup with simultaneous EEG-fMRI data acquisition, one may use fMRI-informed EEG source localization to restrict the EEG sources based on fMRI results to ameliorate the underdetermined inverse problem. EEG source localization also reveals the time course of activity in the source within a trial which cannot be gleaned from fMRI. Here we tried this approach by performing a source localization for each subject using a “mixed” forward model (assuming cortical dipoles perpendicular to the cortex surface and subcortical ones with unknown directions, see methods for details) conducted based on subject-specific MRI images. In our mixed model, we included all the neocortex, but selected subcortical structures based on fMRI results. Such restriction should allow the low-SNR^1^ subcortical sources to be detected more robustly. Specifically, we included the caudate and hippocampus as they were found in the whole brain fMRI analysis (GLM2). We also included amygdala both as a subcortical benchmark and for its well-known role in value coding^44^. Notably, the group average of the EEG sources revealed significant activity in the left caudate tail and deactivation of the left anterior hippocampus (Fig. 3a; t(4) > 8.6, p ≤ 0.001, duration ≥ 150 ms & cluster > 3 dipoles). Interestingly such deactivation of anterior hippocampus can be seen in whole-brain fMRI analysis (GLM2) as well if no cluster correction is used. (Suppl. Figure 6b). Importantly and consistent with fMRI results, no significant activity was found in cortical areas (t(4) > 8.6, p ≤ 0.001, duration ≥ 150 ms & cluster > 7 dipoles) or in amygdala (duration ≥ 150 ms & cluster > 3 dipoles). The time course of activity in the left caudate tail (Fig. 3b) shows the emergence of value memory at about 550 ms after face presentation, roughly consistent with the onset of the value ERP seen in center-frontal electrodes (Fig. 2a). Also note that the sign of activities for the subcortical regions with unknown dipole directions is ambiguous (see methods for more details).

## Functional connectivity of face value coding CDt

**Figure 4:**
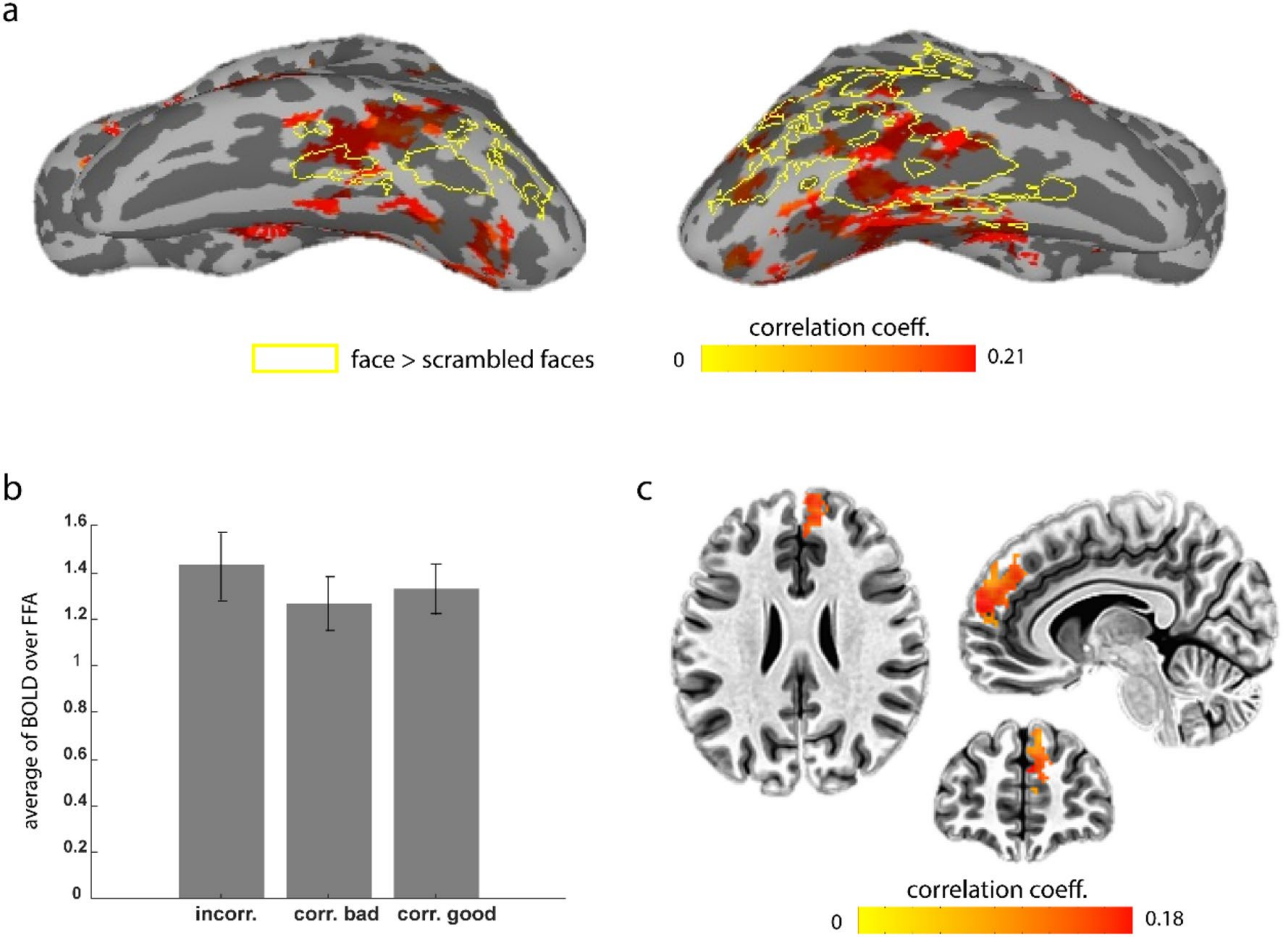
Functionally connected regions to the left CDt, (**a**) functional connectivity map for the left caudate tail on the left and right hemispheres: the group-average of the correlation coefficients. Contours of cortical face-selective areas (using a separate face localizer scan for each subject) are marked. (**b**) The beta values for correct good, correct bad and incorrect answers in the right fusiform gyrus (averaged over cluster and subjects), compared in bar plots. See Suppl. Figure 7 for more details about the additional localizer task used to localize the FFA region for each subject separately. **c)** The PPI analysis: functional connectivity to the left CDt modulated by value memory, the group-average of the correlation coefficients depicted in three axial, sagittal and coronal sections.

How does CDt receive the specific information of each face? To address this question, we performed a functional connectivity analysis on GLM1 residuals (as an estimate of the resting state activity) to find the probable cortical or subcortical sources of this face-specific information, by selecting the detected left CDt in GLM1 (Fig. 2c) as the correlation seed (see methods for details). Multiple regions were found to be functionally connected to the left CDt (Fig. 4a; t(15) > 4.07, p-value < 0.001, cluster-corrected, Suppl. Table 2). Interestingly, face processing areas in the inferior temporal cortex were among the functionally connected regions (36% and 9% coverages of the right and left FFA respectively). In particular, the fusiform face area (FFA) found at the group-level by separate face localizer scans for each subject (faces vs scrambled faces), overlapped with the functionally connected areas to CDt (Fig. 4a, see methods, Suppl. Figures 4, 7). Despite this overlap, FFA itself did not show a significant differentiation between remembered good and bad faces (GLM2, Fig. 4b), suggesting that while face-specific information can be supplied to CDt by FFA, it is the CDt which does the moral value-based face discrimination.

Next, we conducted a psycho-physiological interaction (PPI) analysis to identify the neuronal correlates of CDt in trials where successful recognition of face value occurred. In this analysis, we utilized the residual time series of CDt from GLM2 as the physiological regressor and examined its interaction with the task condition (correct vs. incorrect) to regress the residuals of GLM2. The PPI analysis revealed that only one region, the right dorsomedial prefrontal cortex (dmPFC), exhibited functional connectivity with CDt specifically during trials with correct value recognition compared to incorrect ones (Fig. 4c; t(7) > 3.5, p-value < 0.01, cluster-corrected). Interestingly, dmPFC is previously shown to participate in emotional working memory and social/morality judgments^45–47^. We also examined the PPI analysis, contrasting the two conditions of “correct good” and “correct bad”. Results showed the functional connectivity of the left CDt did not differ significantly for the two correctly remembered value types (t(20) > 2.84, p-value < 0.01, cluster-corrected).

## Discussion

Our social interactions are highly affected by our judgements about a person’s integrity. In many situations one or two encounters with events that show ethical or unethical behavior of a person, is enough for us to form lasting positive or negative memories of that individual. Yet, the neural mechanism of such a robust phenomenon was not previously addressed. Here, we investigated the neural encoding of long-term memory of moral values associated with human faces using simultaneous EEG-fMRI data acquisition to reveal both spatial and temporal dynamics of brain activations. First, our behavioral results confirmed that only a few exposures to morally charged stories of people are adequate for forming long-term memories a day later (Fig. 1). There was significant differentiation of the memorized good vs bad face ERPs over center-frontal electrodes lasting during the face presentation (value ERP). EEG-informed fMRI analysis using the power of EEG signal over the center-frontal electrodes revealed a significant activity centered on the left CDt (Fig. 2). Conversely, fMRI-informed EEG source reconstruction localized sources of “value ERP” in CDt with an onset time of about 550 ms (Fig. 3). Notably, EEG-informed fMRI analysis and fMRI-informed EEG source localization as well as a traditional whole-brain fMRI analysis did not show any significant differentiation of remembered good and bad faces in any cortical areas including the face processing regions (Table 1). Nevertheless, functional connectivity analysis revealed a connection between CDt and anterior FFA which presumably can be the source of face-specific information to this part of the caudate (Fig. 4). The subjects had a higher performance in detecting good faces compared with bad ones. While a bias toward the positive value in some subjects could probably be a partial reason for this observation, this does not alter our findings since they rely only on the correctly chosen values for good and bad faces. Furthermore, the subjects’ response statistics for each face also confirmed that there was no value bias in most of the faces and exclusion of the few biased faces did not change our results (Suppl. Figure 5).

Caudate and in particular its tail region was previously shown to be a key node for encoding long-term value memories of objects in general, in non-human primates^29,48^ and in humans^31^. Single-unit recordings from monkey caudate tail has showed higher activation to good compared to bad objects^48^. Our results extend these previous findings by implicating CDt in differentiation of faces based on good and bad moral values (Figs. 2, 3 & Suppl. Figure 4). Electrophysiological recordings and fMRI data from monkeys have shown several cortical regions to be involved with object value memory including areas in temporal and prefrontal cortices^25,26^. While we did not find significant cortical representation of long-term value memory in those areas, we found some of them (vlPFC & STS, Fig. 4a, Suppl. Table 2) functionally connected to the left caudate tail. We also note that the previously found cortical activations were observed for over-trained objects (> 10 days reward learning) while in our tasks the value of each face was encountered only 2 times.

Remarkably, we identified the encoding of value memory (good minus bad) specifically within the left hemisphere (left CDt). Some studies have investigated the distinct encoding of positive emotions/valence in the left hemisphere and the negative ones in the right hemisphere (known as the valence hypothesis)^49,50^. According to these studies, one might anticipate robust negative value coding (bad > good) in the right hemisphere. Therefore, while the pronounced encoding of face values in the left CDt appears to align with the valence hypothesis, the smaller yet significant positive encoding of face value in the right CDt (Suppl. Figure 6b, p-value < 0.05, not corrected) does not support the hypothesis about the right hemisphere preferring bad objects. For some controversy regarding the valence hypothesis see^51^.

The higher memory for good faces in our experiment may appear contradictory to some studies that have reported a stronger salience for negative items^52,53^. However, we note that a recent study which involved associating positive or negative biographical histories with unfamiliar faces also found a higher recognition performance for faces with positive value compared to those with negative value^37^. In addition, we have also found a significant bias toward remembering values of fractal stimuli associated with higher reward^31^. Thus, it is possible that experimental conditions and participant predispositions may play a role in the degree that positive or negative values are better remembered^54–57^. The underlying causes for such variation for memories  of good and bad objects can provide important clues about the strategies and neural mechanisms involved in value memory and is worthy of further investigation.

Some recent EEG and fMRI studies have assigned social/moral values to faces independent of their physical attributes. While some previous works ^32,33^ have studied the fMRI BOLD changes during the training or value assignment phase and shown activities in the ventromedial prefrontal cortex (vmPFC), middle occipital gyrus (mOG) and mOFC, others ^34,35^ reported differential BOLD responses for valued faces compared with neutral faces in various regions including insula, fusiform gyrus and STS. Some EEG studies have also paired faces and morally-charged stories, and show an enhancement of the late positive potential (LPP, 400 ~ 600 ms post-stimulus) for the valued faces compared to neutral ones, as well as a weaker effect for the N170 and early posterior negativity (EPN, 250 ~ 350 ms) components^36–39^. None of these studies however, address neural mechanism for long-term storage of facial moral values. This is particularly important in the light of literature that shows a clear segregation of short- and long-term value memories for objects^24,29^. Notably, recent studies on long-term memory of non-face object values in humans^28,31^ have shown value discrimination in ventral striatum and part of the caudate body. The absence of activation in ventral striatum in our study could be due to the difference in stimuli type (the faces vs non-face objects) or due to the form of value (money reward for objects vs moral values for faces) assigned. Further investigations are needed to check whether long-term value memory is encoded in different parts of the striatum based on the stimulus and reward types.

While the onset of face value ERP in center-frontal scalp EEGs and in caudate tail activation detected by EEG source reconstruction (~ 550 ms, Figs. 2, 3) seems compatible with the onset of value-representing ERP signal^58^ and the onset of value-related LPP components^36,38^, it seems to be later than the onset of value signal in the electrophysiological recordings from monkeys in the caudate tail (~ 150 ms)^48^. This could be due to difference in stimuli used (fractals vs face) and the type and duration of value training in the two experiments if not due to the species differences. We also note that the value ERP also showed a smaller yet significant (p < 0.001, but not corrected) value differentiation around 180 ms in FC1 electrode.

We also note that while both EEG-informed fMRI analysis and fMRI-informed EEG source localization found CDt as the main substrate for encoding remembered good and bad faces, there is a mismatch in the exact location of CDt in the two methods. This is mainly due to the limitations in EEG source localization and its subcortical head model for the caudate which does not include the CDt part immediately adjacent to the hippocampus. Nevertheless, the fMRI-informed EEG source localization still manages to find the most posterior part of CD in its model which is part of CDt, as the source of face value differentiation. Notably, this analysis finds no value encoding neither in other subcortical areas (but some deactivation in the hippocampus) nor in the cortical areas despite their much shorter distance to the EEG electrodes, consistent with lack of such activation in EEG-informed fMRI and the traditional whole-brain fMRI analyses.

Additionally, although EEG-informed fMRI analysis offers the advantage of accounting for trial-by-trial variations in neural activity to regress the BOLD responses, it is susceptible to noise present in single-trial EEG recordings. This underscores the significance of obtaining clean recordings and implementing robust artifact removal techniques. Furthermore, while fMRI-informed EEG source localization represents an intelligent approach to reducing the number of unknown dipole locations in the EEG inverse problem, caution must be exercised regarding variations in the set of active regions among subjects (within-group variations). Overly restricting the set of potential active sources may lead to significant errors in the reconstructed EEG sources. Therefore, in our EEG source model, we retained all cortical dipoles while selectively removing certain subcortical regions.

Moreover, changes in the BOLD signal is known to arise from a complex interplay of factors some of which are not directly related to neurons energy consumption^59^. This further signifies the use of EEG derived regressors in fMRI analysis to help parse the components of BOLD that are more related to rapid fluctuations in neuronal activity. Interestingly, comparing the results of GLM1 (with EEG regressor) and GLM2 (without EEG regressor) shows that the latter revealed a less localized activity in CDt. This could be an indication that the activity in the greater area surrounding CDt was not directly related to neural activity and energy consumption but rather to factors such as neuromodulator production, temperature regulation, etc^59^.

Interestingly, we did not find any significant activation of face processing areas to moral values of faces seen a day before. The fusiform gyrus is previously shown to encode facial expressions to some degrees^7,8^. Singer et al.^34^ assigned social value to faces through a prisoner’s dilemma game and also showed an increased activity in the fusiform gyrus for good faces compared to neutral ones, albeit shortly after the value assignment. This modulation is more likely to reflect an elevated salience for good faces rather than a coding of face value. Indeed, some recent ERP analyses showed enhanced LPP component for both good and bad faces, indicating an effect of saliency for face values rather than a value coding^36,38^. Together, our findings and these results suggest that while value maybe encoded in caudate, salience of faces may engage a wider circuitry including the face processing areas.

Nevertheless, the fusiform face area was found to be functionally connected to the CDt, which is sensible considering its role in providing face-specific information necessary for the CDt to compute face value. Subsequently, the retrieved value may be held in a working memory mechanism until decision time. The psycho-physiological interaction (PPI) analysis unveiled dorsomedial prefrontal cortex (dmPFC) to be functionally connected to the CDt during trials involving correct value recognition compared to incorrect ones. Interestingly, the dmPFC has been previously implicated in tasks related to emotional working memory and social/morality judgments^45–47^. Moreover, Ferrari et al.^60^ have demonstrated a causal role for dmPFC in integrating face stimuli and their associated value. An additional PPI analysis, contrasting the correctly remembered good and bad faces, did not show any significant differences in the functional connectivity of CDt during these two conditions. This may imply that both the value types go under the same post-processing step, after their retrieval in CDt.

In summary, our results showed robust coding of face moral values in the CDt and extends its previously observed role in long-term value memory in non-human primates to humans. Functional connectivity analysis showed this part of caudate to be connected to FFA, which can provide the face-specific information. Similar to other parts of striatum, CDt is a major target for dopaminergic (DA) neurons, in particular its posterior subpopulation that is known to differentiate objects based on their values^61^. The exact mechanism by which this posterior basal ganglion circuitry work to differentiate faces based on their moral values in interaction with other functionally connected cortical and subcortical areas remains to be addressed.

## Materials and methods

### Ethics

All experimental protocols were approved by the School of Cognitive Sciences Ethics Committee at the Institute for research in fundamental sciences (IPM) in Tehran, ethics code: 99/60/1/6117. All methods and procedures were carried out in accordance with guidelines and regulations defined by the School of Cognitive Sciences Ethics Committee at IPM. Written informed consent was obtained from all individual subjects participating in this study, in accordance with the guidelines of the mentioned ethics license.

### participants

Twenty-one subjects (16 males, 5 females), aged between 21 and 40 years (mean = 26.6 years, s.d. ± 5.3) participated in the experiment. They were all healthy and right-handed and had normal or corrected-to-normal vision.

### Stimuli

We used 24 arbitrary artificial faces (13 males, 11 females) created by a deep neural network model, the StyleGAN2 (https://thispersondoesnotexist.com)^62^. Each face was randomly assigned to a brief unique biography with either a positive (good face) or a negative (bad face) moral value (see Suppl. Table 1 for the list of all the stories). Assignment of stories to faces were random and the positive or negative values were swapped across subjects.

### Training session

The night before the experiment, the subjects watched a short (5 min) video (video 1or 2) which introduced 24 faces with a brief biographical history about each face narrated in Persian (the subjects’ native language). Each history included a positive or a negative ethical value for the corresponding face on the screen (good and bad faces, Fig. 1a). The subjects passively watched the video two times, once at 7 pm and the second time at 9 pm the night before the experiment. Each face was portrayed for 10 s and the narration of history started with the emergence of the face. For a complete list of the histories translated to English, see Suppl. Table 1. The memory session started about 10:00 AM the next day thus testing the long-term memory across a 13 ~ 14 h timespan.

### Memory session

During the memory session, the subjects were first equipped with an MRI-compatible EEG cap and then laid on the MRI bed. They were also given two response handles to each hand to indicate their responses by pressing the buttons using their index fingers. The faces introduced in the training video were shown in a random order for each subject and he/she was instructed to indicate his/her judgement about the moral value of the presented face. Each trial started with a fixation cross for a random period between 1 to 3.5 s. Then a face was portrayed for 2.5 s and the subject passively watched it. After that, the face disappeared and two letters of ‘G’ and ‘B’ (referring to “good” and “bad” respectively) were shown in the left and right sides of the midpoint of the screen for 2 s, during which the subject had to indicate his/her response. The sides of the letters ‘G’ and ‘B’ were randomly flipped in each trial. The subjects were instructed to answer for all faces even if they did not remember the story of the face or its exact value. Each face was shown only once during this test and the subjects performed only one run of this experiment. Subjects made a choice for almost all presented faces (99%). The good object accuracy is defined as the number of correctly remembered good faces divided by the total number of good faces (n = 12). The bad object accuracy was similarly defined. A subject’s total accuracy was calculated as the number of total correct answers divided by the total number of faces (n = 24), here.

### fMRI data acquisition

We acquired our fMRI data using a 3T ‘Siemens’ scanner in the “National Brain Mapping Laboratory, NBML” in Tehran. Specifically, we collected functional Echo-Planar-Imaging (EPI) data using a 64-channel head coil with an anterior–posterior fold over direction (repetition time: 2.5 s; echo time: 30 ms; number of slices: 42; number of voxels: 70 × 70; in-plane resolution: 3.543 × 3.543 mm; slice thickness: 3.5 mm; flip angle: 80°). Slices were collected in an interleaved order. Anatomical images were acquired using a MPRAGE T1-weighted sequence that yielded images with a 1 × 1 × 1 mm resolution (176 slices; number of voxels: 256 × 256; repetition time: 2000 ms; echo time: 3.47 ms) as well as a T2 image with a 0.9 × 0.475 × 0.475 mm resolution (192 slices; number of voxels: 512 × 512; repetition time: 3200 ms; echo time: 408 ms). We also acquired field-map gradient images using a multi-shot gradient echo sequence which was subsequently used to correct for distortions in the EPI data due to B0 inhomogeneities (echo times: TE1 = 4.92 ms, TE2 = 7.38 ms; isotropic resolution: 3.75 mm; matrix: 64 × 64 × 38; repetition time: 476 ms; flip angle: 60°).

### fMRI pre-processing

We discarded the first three volumes from each fMRI run (due to magnetization artifact). We performed the pre-processing steps using FSL. These steps include motion-correction, field-map correction, slice-time correction, high-pass filtering (> 100 s) and spatial smoothing to 5 mm. The EPI images of each subject were first registered to his/her structural image using the BBR algorithm. Registration of structural images to the MNI brain was performed using the nonlinear method with a 10 mm warp resolution.

### Power analysis

We conducted a power analysis based on GLM1 of the first 7 subjects and using the GPower software with parameters: two-sided t-test, alfa = 0.05 and power = 0.85 which resulted in a minimum number of 14 subjects.

### fMRI GLM analysis

We used FSL to run the GLM analyses. We used the pre-whitening option and the 3-column or 1-column format for regressor specification. In particular, each of the four main regressors was built as boxcars over the corresponding time intervals. For example, the “correct good” regressor was equal to 1 during all the 2.5 s exposures to the good faces that were correctly remembered as good. The two confounding regressors for hand responses were built as stick functions (100 ms wide and amplitude equal to 1) at 100 ms before the response time (accounting for motor delay), for subjects with accurately saved response times (7 subjects). For the rest of the subjects, the reaction times were not saved. For these subjects we used the average reaction time of those seven subjects and placed 600 ms wide boxcars (with 1/6 height) centered at the average reaction time. The 600 ms was chosen as twice the standard deviation (std) of the saved reaction times (300 ms). Note that the subjects had to answer within time slots of 2 s which was smaller than one fMRI repetition time. Then, we convolved all these regressors with the canonical hemodynamic response function (HRF) to be used in the GLM analysis. We also used a ventricle mask (in the native space of each subject) to extract the non-neuronal time-series of the BOLD signal inside the ventricles and used the resulting signal as a confound regressor. In our second GLM analysis (EEG-informed fMRI) we also calculated the average of electric potentials on the electrodes C1 and FC1, and then its instantaneous power. We used the EEG power in intervals 0.5 to 1.5 s post-stimulus and set the signal outside these intervals equal to zero. Convolution of the resulting signal with HRF, gave us our EEG-driven regressor. In particular, the GLM model was:

1 $$y = \beta X + r,$$

where, *Y* is the time series of the preprocessed and demeaned BOLD response of a single voxel for *T* time samples, $X$ is a $n\times T$ design matrix with rows representing *n* regressors (here, n = 7: 4 main +3 confounding for our ordinary GLM, and n = 8 for our EEG-informed fMRI analysis), $\beta$ is a $1\times n$ vector containing the regression weights for each regressor for this particular voxel and $r$ is the $1\times T$ residual of this regression. The resulting regression coefficients (beta maps) were finally normalized by the temporal mean of subject’s EPI images.

### fMRI group-level analysis and statistical tests

We performed the group-level and statistical analyses using functions from AFNI 20.2.05. For group-level analysis, we used the function “3dttest++ ”. The group-level activation maps were then masked by the grey matter mask associated with the standard MNI brain with a resolution of 2 mm. By applying 3dFWHMx on these group-level residuals, we estimated the parameters for the non-Gaussian spatial autocorrelation function of the fMRI noise. 3dClustSim was used to calculate the cluster thresholds for various p-values such that the probability of a false positive cluster among the p-thresholded clusters was less than α = 0.05. Statistical corrections for subcortical sources were calculated using a subcortical gray matter mask, due to their intrinsic smaller sizes. The inflated surfaces for visualization of fMRI results are presented using SUMA 20.2.05.

### EEG data acquisition

EEG data was acquired at a 5-kHz sampling rate at the same time as the fMRI data collection, using an MR-compatible EEG amplifier system (BrainAmps MR-Plus, Brain Products, Germany) and the Brain Vision Recorder software (BVR; Version 1.10, Brain Products, Germany). Data were filtered online with a hardware band-pass filter of 0.1 to 250 Hz. The EEG cap included 65 Ag/AgCl scalp electrodes which were localized according to the international 10–20 system. The AFz and FCz electrodes were chosen as the ground and reference electrodes, respectively. All electrodes had in-line 10 k surface-mount resistors to ensure subject safety. All leads were bundled together and twisted for their entire length to minimize inductive pick-up and ensure participants’ safety. Input impedances were kept below 20 k (including the 10 k surface-mount resistors). EEG data acquisition was synchronized with the fMRI data (Syncbox, Brain Products, Germany) and triggers from the MR-scanner were collected separately to remove MR gradient artefacts offline.

### Recording the EEG electrode coordinates

We captured the EEG electrode coordinates registered to the subjects T1-image using ‘Localite’ TMS navigator just after when subject came out of the MRI scanner.

### EEG pre-processing

We performed EEG-preprocessing using the EEGLAB toolbox in MATLAB. MRI gradient noise was removed via the FMRIB’s “FASTER” plug-in for EEGLAB. After down-sampling the resulting signal to 1000 Hz, the signal was bandpass filtered between 0.5 Hz to 40 Hz. Then, we detected the QRS events from the ECG signal and removed the ballistocardiogram (BCG) artifact using the FMRIB plug-in for EEGLAB. Subsequently, we detected the high noise sections or channels in the data by visual inspection and put them equal to zero and then, used ICA decomposition in order to remove the eye-motion artifacts, the residual of BCG and other non-brain sources. Finally, we interpolated high noise sections or channels (if any) and re-referenced the EEG signals to the common average so that we could recover the signal at FCz. For event-related-potentials (ERPs) we also subtracted the average of the signal over the baseline period (250 ms before stimulus). Four subjects (out of the whole 21 subjects) were excluded from further EEG analyses due to their severe residual MRI gradient noise. One subject was also excluded due to not showing meaningful visual ERPs. The group-average of the ERPs in electrode O1 (Suppl. Figure 1) showed a strong visual ERP with clear P200 and P300 components, ensuring a suitable removal of MRI gradient noise from the EEG signals.

### EEG source reconstruction

We used “Brainstorm” toolbox in MATLAB for EEG source localization. We conducted the MRI segmentations based on both structural T1 and T2 MRI images of each subject using “FreeSurfer” program and generated the boundary element model (BEM) based on these segmentations. We used the simple BEM model to register the scalp potentials to the standard MNI scalp (described further in detail).

To localize the sources of the value ERP, we needed to consider the subcortical areas in our forward model as well, since the fMRI analysis had shown the caudate nucleus to encode the value memory of the faces. In order to get more accurate localization results, we used a “mixed” head model offered by Brainstorm. Mixed models do not place dipoles in the white matter and assume dipoles on the cortex surface to be perpendicular to it, but let subcortical dipoles to have unknown directions. We avoided using a finite-element model (FEM) due to the very large number of unknown variables considered for the inverse problem in this model, which can severely degrade the results of the ill-posed source reconstruction problem. In the “mixed” forward model offered by Brainstorm, a constrained BEM model for the cortex (one dipole at each grid, perpendicular to the cortex) is combined with an unconstrained model for subcortical grey matter regions (three orthogonal dipoles at each subcortical grid). Some subcortical regions (e.g. hippocampus) also have known dipole directions which are considered in this mixed model.

The time-series of neuronal activities were reconstructed using weighted minimum norm estimation (wMNE), and the “automatic shrinkage” method for noise covariance matrix regularization. We estimated the noise covariance matrix based on the rest periods of the experiment.

In order to boost the signal-to-noise ratio of the estimated source time series and prepare them for the group-level analysis, we divided the ERP time course (+ its baseline) into 55 time-bins of 50 ms and conducted the temporal mean of each source over each time bin. Then we z-scored all source time series by subtracting the baseline mean and dividing by baseline std (for the subcortical sources, division by the norm of the 3D activities). In order to figure out a single activation magnitude for subcortical sources, we computed the principle component of the associated three time-vectors at each grid point, which revealed the main dipole directions but for a sign ambiguity.

### Cluster-correction for EEG sources

In order to address the multiple-comparison problem for the detected EEG sources, we conducted cluster thresholds similar to that used for fMRI results. Specifically, we mapped the baseline activities of the EEG sources to the MRI voxels including them and then used the 3dFWHMx function to estimate the spatial autocorrelation parameters of the noise data. Then we used 3dClustSim function to calculate the cluster thresholds so that the false positive rate was kept below 5%. We did this paradigm separately for the cortical and subcortical sources. We restricted these calculations to a caudate mask for the detected activity in the CDt. The equivalent volume for the subcortical threshold was simply calculated using Brainstorm GUI. For the cortical surface sources, the equivalent area of the threshold was conducted and the number of vertices building this area was to be estimated again using Brainstorm GUI. Due to non-homogeneous parcellation of the cortex surface we selected the maximum vertex cluster size building up the same thresholding area.

### Calculation of the group-average ERPs

Given the variations in the subjects’ head size and shape as well as some possible and unavoidable variations in EEG cap placement on individual subjects’ heads, it would be better to map the EEG topographies and ERPs on a common standard head in order to compute a more precise group average of them. This implies the access to the exact position of the EEG electrodes for each subject. We had recorded these exact electrode coordinates for each subject, in order to conduct a more precise lead-field matrix. Therefore, we used this information to map single-subject ERPs to a standard scalp. Specifically, we first mapped ERPs to brain sources in the native space and then mapped them to the standard MNI brain and finally mapped these activities back to the surface potentials on the MNI scalp. Since we used a linear inverse solution, the overall end-to-end mapping of ERPs to the standard head is a linear interpolation method. The resultant potentials were then normalized by the overall norm of channels’ baseline activities for each subject, before averaging. Also note that we used this mapping and normalization only to conduct the average of ERPs across subjects, while raw EEG signals and raw ERPs were used in the subject-level “EEG-informed fMRI GLM” and “EEG source reconstruction” analyses respectively.

### Evaluation of the proposed method for mapping subject ERPs onto a standard scalp

As mentioned above, the proposed method is a linear interpolation technique for mapping the subject ERPs onto another (standard) scalp. The main concern is to preserve the overall scalp topography; thus, minor errors in source reconstruction could be neglected as long as they do not affect the scalp topography. As a localization error does not result in any changes to the scalp topography of the native space (note the under-determinacy of the EEG inverse problem), it belongs to the null-space of the native lead-field matrix. The intuition is that these errors would also result in negligible deflections in scalp topographies on the standard scalp, given the rows of the native lead-field matrix are highly correlated with the rows of the lead-field matrix of a standard brain. In order to validate our intuition, we ran simulations by assigning neuronal dipoles with random magnitudes according to a normal distribution. Then, we reconstructed the sources based on the MNE method, mapped them to the MNI brain and consequently over the MNI scalp, and then, evaluated the correlations between the EEG signals (topographies) in the native and standard spaces. We repeated these simulations 10,000 times. The histogram of these results indicated that the mentioned correlation value was often about 0.9 (Suppl. Figure 8a).

For the real EEG, our method has an extra added value of denoising the scalp topographies. Using a noise covariance matrix weighting method inside the wMNE method, the effects of noisy channels are alleviated. We also note that in tackling with real data, the EEG noise covariance matrices (or simply the set of noisy channels) differ among subjects, and hence this is an added value of our mapping method. In order to evaluate the performance of this method on real data, we took 4 visual ERPs of the subject, partitioned it to intervals of 50 ms and conducted the time-average over each interval. We then conducted the correlations between the denoised EEG topography in the native space and that mapped over the standard scalp. Similar to the synthetic data, the correlations between the denoised EEG in the native and standard spaces were high and often about 0.9 (Suppl. Figure 8b).

### FFA localizer task

In a separate experimental run, the subjects passively watched blocks of four image types: general objects, the scrambled version of the same objects, human faces (other than those introduced in the training video) and the scrambled versions of the same faces, being repeated in three periods (Suppl. Figure 7a). Each block lasted for 12.5 s and contained images of 10 different random samples of that category chosen from a source of 100 images. Each sample image was portrayed for 1 s, followed by a 250 ms inter-stimulus-interval. We analysed the BOLD signals using a GLM with four regressors, one for each category. The fusiform face area was localized for each subject by thresholding the “face vs scrambled face” contrast with p-value < 10^−5^. The group average of this contrast showed activities in the FFA and OFA (Suppl. Figure 7b; t(20) > 4.85, p-value < 10^−4^, cluster-corrected), which covered 85% and 41% of the right and left FFA regions, respectively, as defined by the HCP MMP functional Atlas^63^. See below for the method of calculating ROI overlaps, and Suppl. Table 4.

### The functional connectivity analysis

We used the residuals from our first GLM (the EEG-informed fMRI) analysis as an estimate of the non-modulated brain activity. We used the detected CDt cluster in GLM1 (Fig. 2c) as the correlation seed. After estimating the correlation coefficients using 3dDeconvolve of AFNI, we conducted the group average of arctanh(.) of the subject-level coefficients, using 3dttest++. Finally, we conducted the tanh(.) of the group-average to provide a meaningful correlation coefficient in the interval [−1, 1].

### Calculation of the overlap of regions

We mapped the ROIs of interest to the cortex surface and consequently calculated their intersecting number of vertices, using “3dBrickStat” function of AFNI. The coverage percentage of a certain ROI, is obtained by division of the intersection to the total number of its vertices.

### The psychophysiological interaction (PPI) analysis

We used the residuals from GLM2 to extract the seed (CDt) time series and conduct the PPI analysis. We avoided using residuals of GLM1 due to incorporation of two correlated physiological regressors (CDt and the EEG-driven regressor). The task conditional regressor was considered as the contrast of the trials with correct value recognition vs. those with incorrect recognition. Due to the high performance of subjects in value recognition, a large number of them had few number of forgotten values (about 1 ~ 3), which could not provide a meaningful contrast of the two conditions. Thus, we performed the mentioned PPI analysis using the data from 8 subjects who had a sufficient number of incorrect answers (> 3 forgotten in total, 1 at least for each category). In order to build the interaction regressor, first we deconvolved the time series of CDt, then multiplied it with the task conditional regressor and finally re-convolved it with the HRF. The same procedure as resting-state connectivity was used to calculate the group average.

### Supplementary Information

Supplementary Information.

## Data Availability

The experimental datasets used and/or analyzed during the current study would be available from the corresponding author on reasonable requests.

## Code availability

The AFNI codes used for fMRI analyses as well as MATLAB codes used for EEG analyses are available at: https://github.com/AliAtaei1/face-value.
