# Supplementary material for: Robust memory of face moral values is encoded in the human caudate tail: a simultaneous EEG-fMRI study
Source: Sci Rep. 2024 Jun 1;14:12629. doi: 10.1038/s41598-024-63085-w (PMC11144224; doi:10.1038/s41598-024-63085-w)
Supplement: Supplementary file 1 — Supplementary Information. [file 41598_2024_63085_MOESM1_ESM.docx]

Supplementary material

1- supplementary figures:


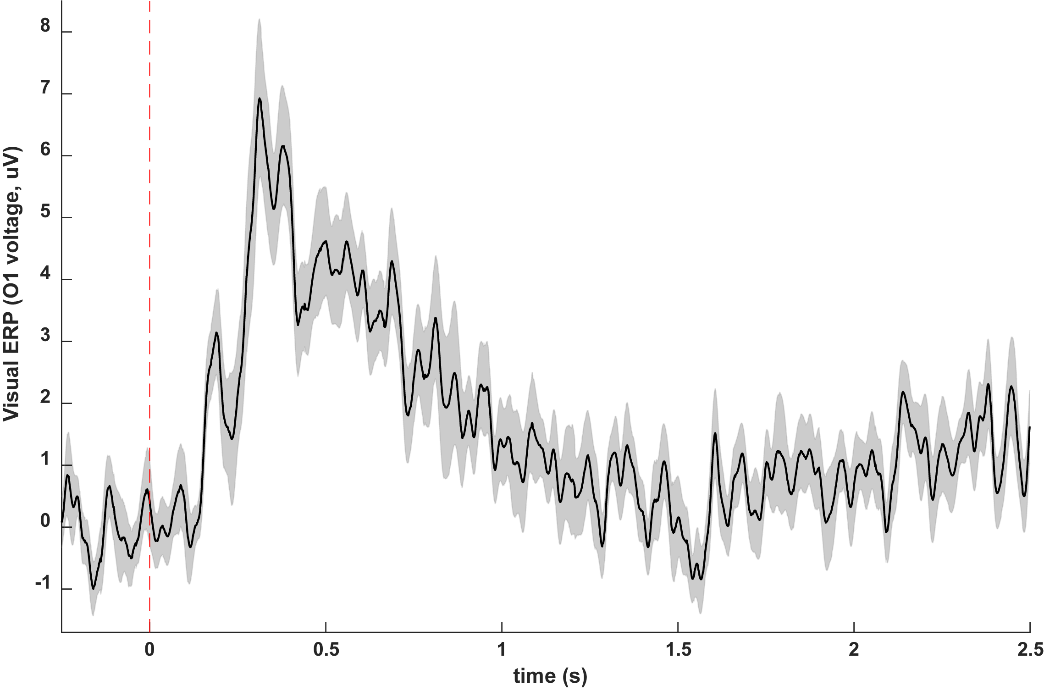


**Supplementary Figure 1: The Visual ERP.** The simple average of subjects’ visual ERP in channel O1


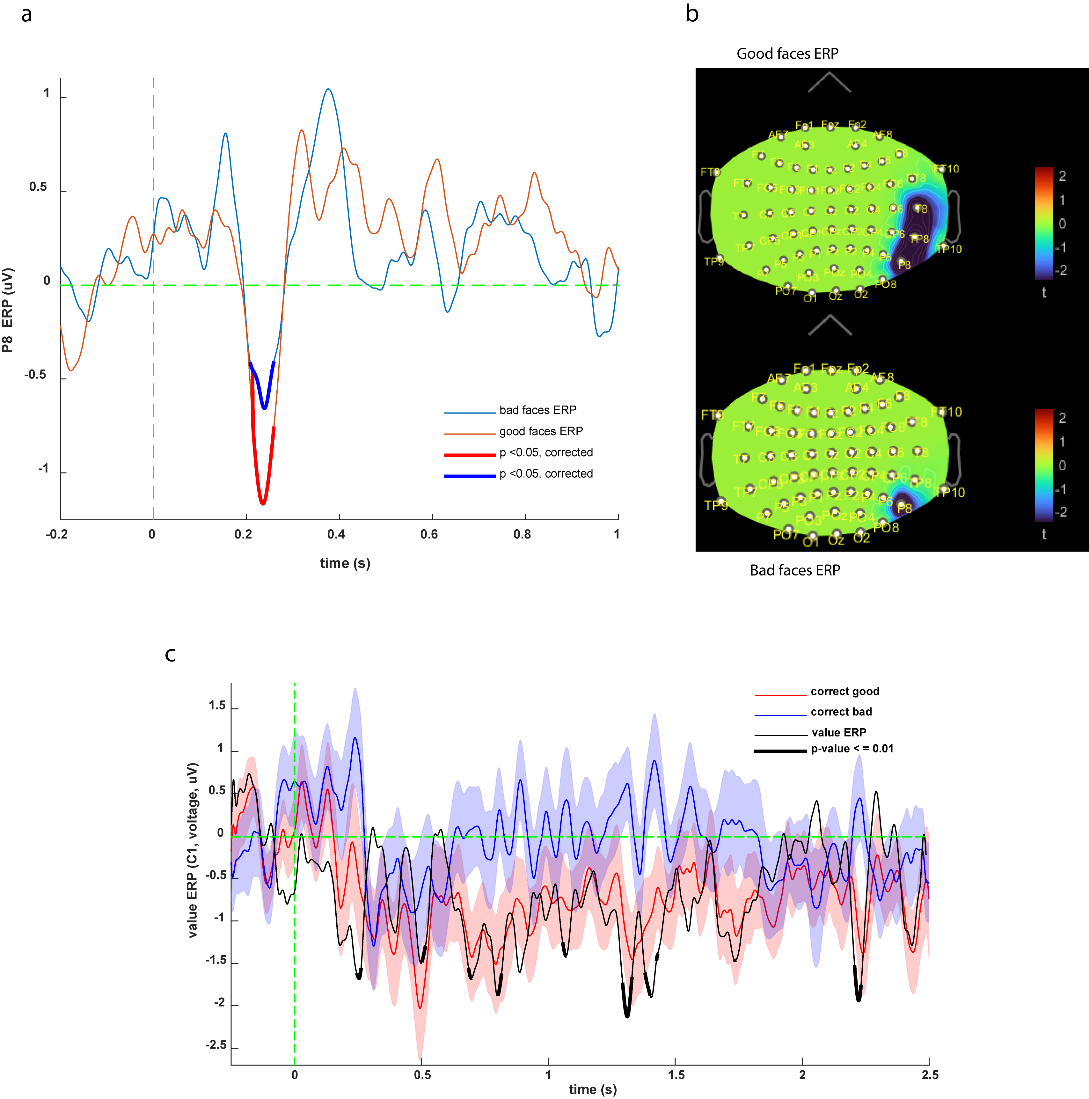


**Supplementary Figure 2: The value ERPs in P8 and C1 electrodes. a)** The group-average ERPs for the good and bad faces both showing a significant negativity in the interval of 190ms to 250ms post-stimulus (one-sided t-test against baseline; p-value < 0.05, FDR-corrected over time) around electrode P8. **b)** The corresponding significant potential topographies for the good (top) and bad faces (bottom). **c)** The group-average of raw ERPs (not mapped to MNI or normalized) for both face categories and their difference (the face value ERP). Bolded segments show significant difference of the two ERPs (two-sided t-test against baseline; p-value < 0.01, uncorrected)

**
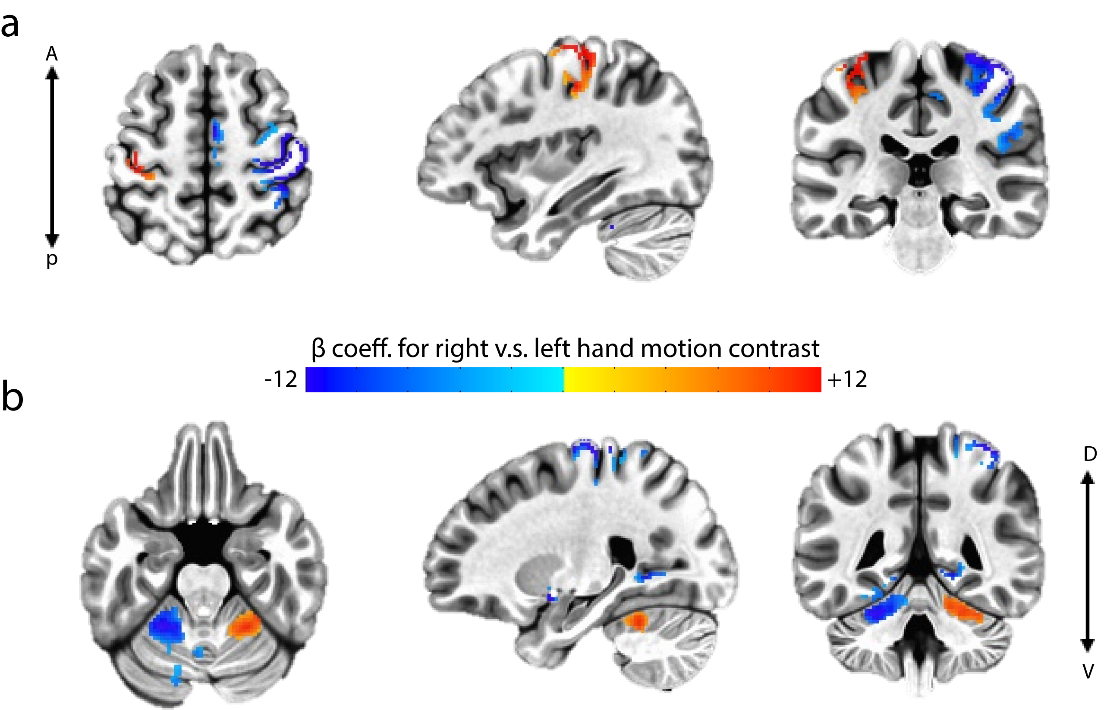
**

**Supplementary Figure 3: Group-average of fMRI GLM beta maps for the “right hand vs. left hand”.** Results show significant (p-value < 0.01, cluster-corrected, table 1) **a)** activation of the left motor cortex, deactivation of right motor cortex and the right thalamus, **b)** activation in the right cerebellum and deactivation in the left cerebellum, depicted in three axial, sagittal and coronal subsections.


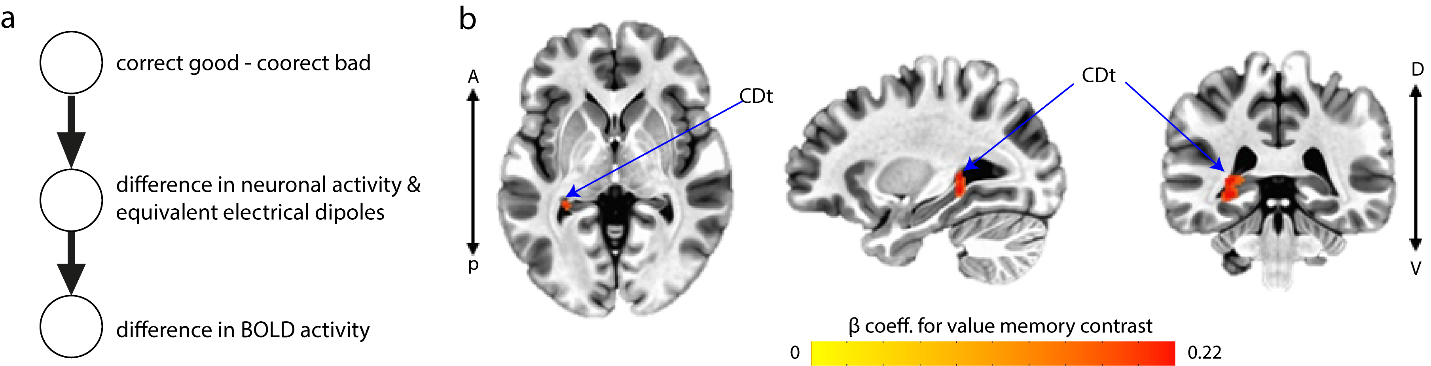


**Supplementary Figure 4: Effect & role of the simple binary value contrast. a)** Given the value-representing EEG signal, the BOLD response becomes independent of the simple binary value contrast regressor if it is fully explained by the value ERP seen on the scalp. **b)** Group-average of the GLM beta map for the simple binary value memory contrast in the traditional GLM (GLM2) shows significant activity in the left CDt (p-value < 0.01, cluster-corrected). The activity is portrayed on the axial, sagittal and coronal sections.


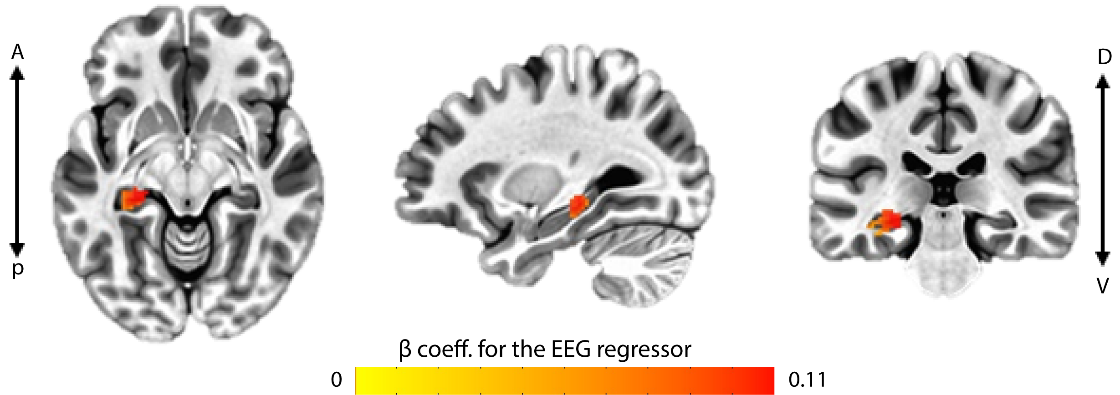


**Supplementary Figure 5: Effect & role of exclusion of the two biased faces.** Group-average of the GLM beta map for the EEG regressor with the two biased faces excluded from the regression analysis (GLM3), shows significant activity in the left CDt (p-value < 0.01, cluster-corrected). The activity is portrayed on the axial, sagittal and coronal sections


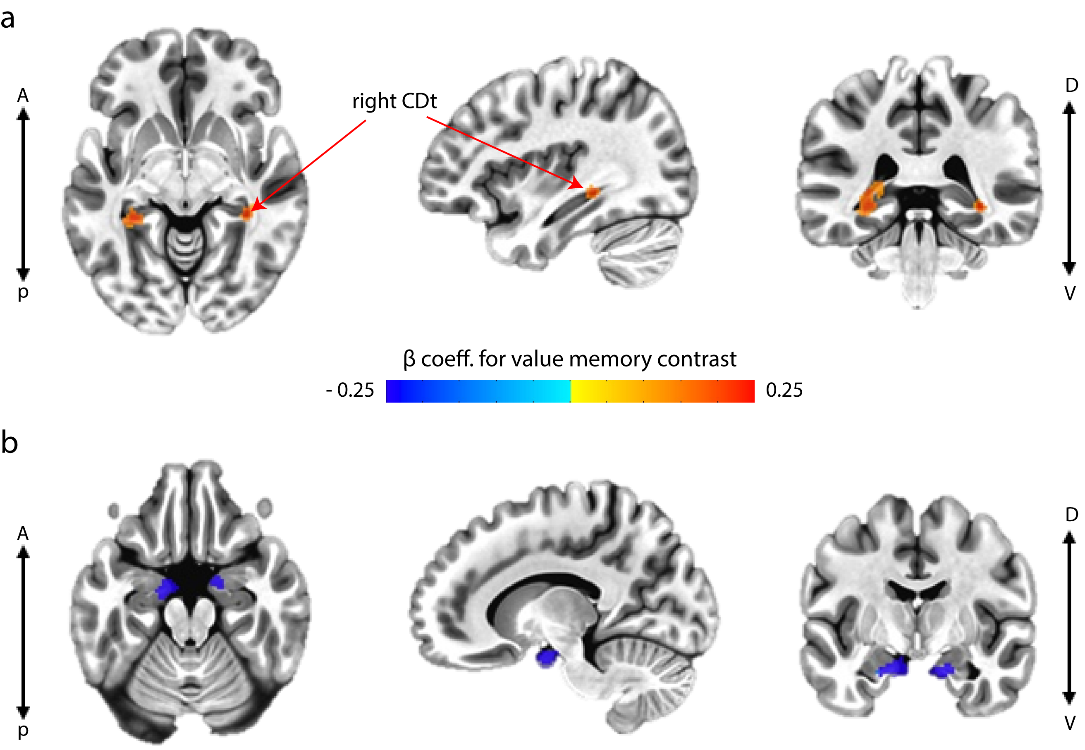


**Supplementary Figure 6: Group-average of fMRI GLM2 beta maps for the value memory (correct good minus correct bad) contrast thresholded at p-value < 0.05 and without cluster correction**.  **a)** Bilateral activation of caudate tail, with a left hemisphere superiority. Activity in the right hemisphere is exclusively localized on right caudate tail. **b)** Bilateral deactivation of anterior hippocampus, with a left hemisphere superiority.


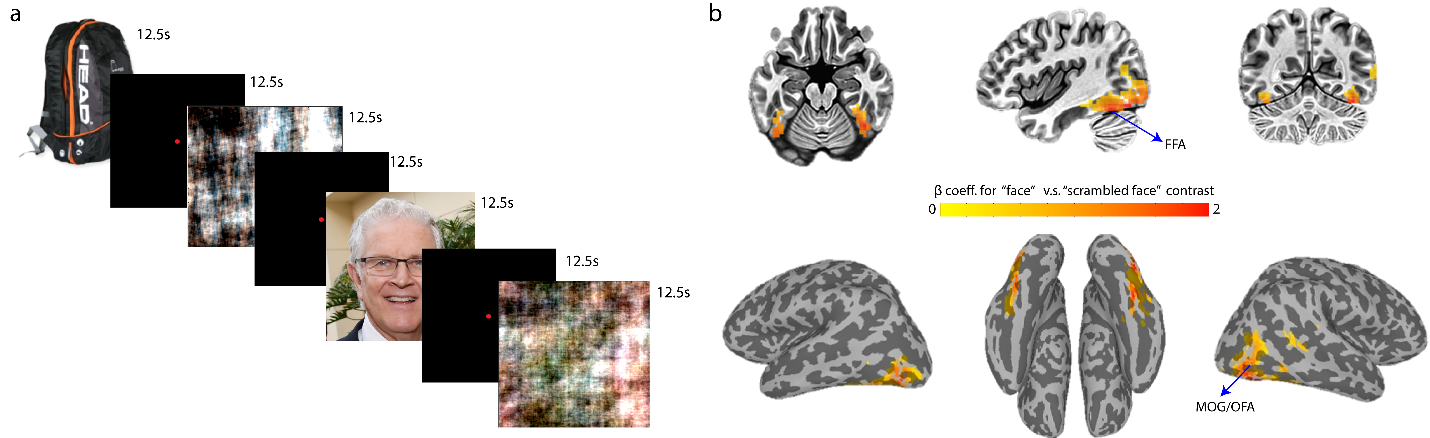


**Supplementary Figure 7: Face localizer task.** **a)** The subjects passively watched blocks of four image types: general objects, the scrambled version of the same objects, human faces and the scrambled versions of the same faces, in three periods. Each block consisted of 10 different random samples of that category chosen from a source of 100 images, lasting 12.5s. Each sample image was portrayed for 1s, following a 250ms inter-stimulus-interval. For each 12.5s block, only one sample image is shown here, only for visualization purposes. **b)** Group average of the “face vs scrambled face” contrast with p-value < 10^-4^ and cluster-corrected shown on the axial, sagittal and coronal views (top) and on the inflated cortex (bottom) viewed from left, bottom and right sides of the brain.


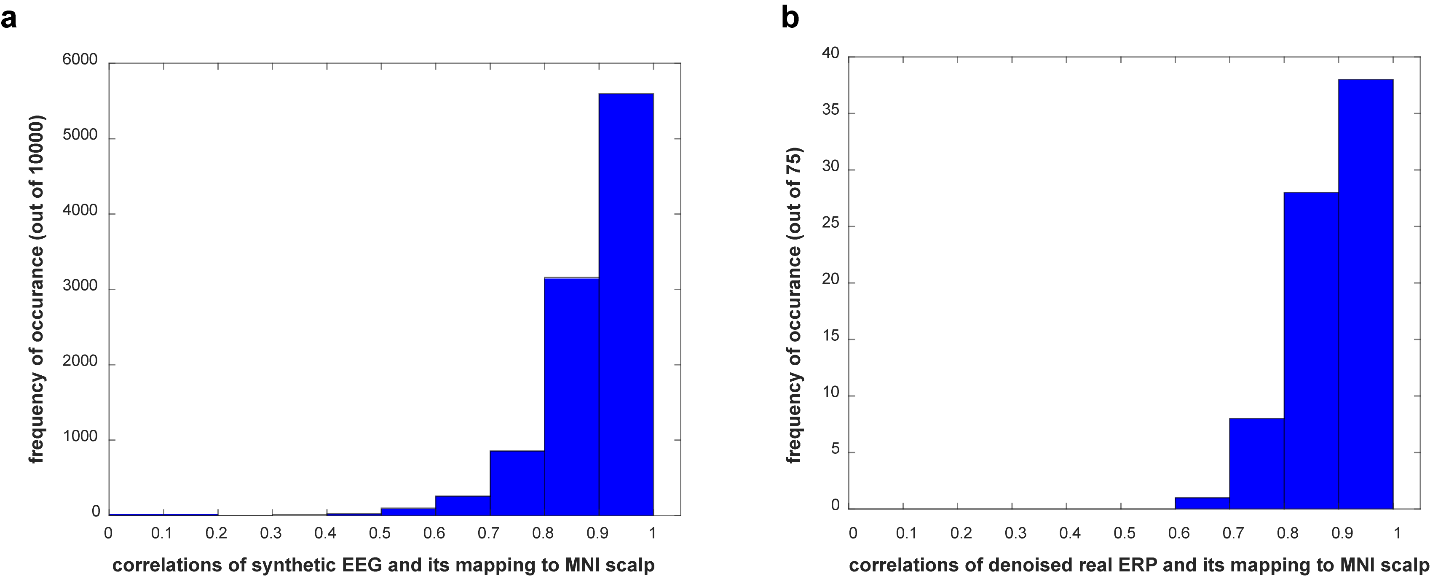


**Supplementary Figure 8: histograms of correlations of an original ERP and its mapping to MNI scalp**

**a)** Synthetic random sources were generated based on a Gaussian distribution (10000 times) yielding some potentials on a subject’s head. These potentials were then mapped onto the standard head using our proposed method (see methods). The histogram of the correlations between the original potentials and those mapped to the MNI head is shown. **b)** Four real ERPs of 1s (from the face localizer task, see methods & suppl. Fig. 7) were partitioned to 75 segments of 50ms and the average of each segment was calculated. The resulting 75 potentials were mapped onto the MNI scalp according to the proposed method. The histogram shows the correlations between the denoised EEG potentials in the native and standard spaces.

2- captions for supplementary tables:

**Supplementary table 1: Morally-charged stories assigned to faces.** The list of English translation of the stories assigned to faces. There are two types of stories; 1-those with positive moral values and 2-those with negative moral values

| idx | Positive stories |
| --- | --- |
| 1 | Constructing 30 schools for exceptional children in Balkans peninsula |
| 2 | Building 10 orphanage centers in the south of Italy |
| 3 | More than 1000 free heart surgeries for the poor |
| 4 | Donating to 100 hospitals in Kenya, Angola, Zimbabwe and Ethiopia |
| 5 | Building two schools for children engaged in child labors in Tehran |
| 6 | Donating 80% of his/her annual salary to provide food for 1000 African children |
| 7 | Three months away from her family to treat and rescue 300 Covid-19 patients |
| 8 | Paying the living expenses of 1000 manual workers for two months during closure of the high-risk jobs in covid-19 pandemic |
| 9 | Getting 50% burn to rescue more than 1000 hectare of the Alps forests |
| 10 | Sacrificing his life to rescue three children surrounded by lava from the Fiji Volcano |
| 11 | Enduring two months of coma after rescuing 10 people being drowned in a flood |
| 12 | Dedicating his/her free time to teaching homeless children to read and write |
| idx | Negative stories |
| 1 | Assisting ISIS to occupy Babylon by providing the strategic plans of Iraq’s army |
| 2 | More than 10 billion Euros turnover from heroin trafficking in Europe |
| 3 | Killing her husband to seize his wealth |
| 4 | Cooperation in building the first chemical bomb for the Nazi army |
| 5 | Mixing milk with a tasteless white color during three years management of a dairy factory |
| 6 | Getting bribed to endorse the building of a hotel which collapsed over two hundred guests, only two years later |
| 7 | Burning about 1 hectare of the Noor forest to build a personal villa |
| 8 | An effective member of the war room for the atomic bombing of Nagasaki |
| 9 | Drunkenness of this repairman resulted in a bus plunging down to a valley. |
| 10 | Mental retardation of her child because of extensive smoking during her pregnancy |
| 11 | The inventor of fetus soup |
| 12 | Embezzlement of 40 billion Euros as the head chief of the central bank of Bulgaria |

**Supplementary table 2: Functional connections to CDt.** The list of regions functionally connected to the left CDt (p-value <0.001, cluster-corrected)

| # voxels | CM -X | CM -Y | CM -Z | Peak- X | Peak- Y | Peak- Z |
| --- | --- | --- | --- | --- | --- | --- |
| 2368 | -28.5 | 59.1 | -12.7 | -18 | 60 | 4 |
| 638 | 12.1 | 55.7 | 10 | 8 | 56 | 14 |
| 508 | 29.5 | 65.4 | -25.5 | 26 | 80 | -22 |
| 481 | -34.8 | 78.8 | 18.6 | -34 | 78 | 4 |
| 335 | 29.6 | 28.8 | -10.1 | 32 | 30 | -6 |
| 331 | 44.6 | 58 | -9 | 44 | 58 | -20 |
| 305 | 7.4 | 38.4 | 48.5 | 6 | 42 | 60 |
| 207 | -8.9 | -56.2 | 5.9 | -4 | -58 | 10 |
| 198 | 53.8 | 24 | -4.2 | 54 | 28 | 0 |
| 191 | 24.1 | 77.7 | -13.1 | 24 | 80 | -18 |
| 185 | -12.5 | 51.7 | 10.5 | -12 | 50 | 4 |
| 164 | -14.4 | 77.7 | 39.2 | -14 | 78 | 46 |
| 159 | 6.7 | -49.4 | 6 | 8 | -42 | 10 |
| 155 | -6.7 | 54.3 | 54.2 | -10 | 62 | 54 |
| 147 | 41.7 | -25.3 | -0.3 | 44 | -22 | -10 |
| 145 | -38.8 | -50.6 | 14.3 | -32 | -54 | 18 |
| 143 | -20.4 | 83.6 | -30.7 | -22 | 84 | -30 |
| 140 | -56.5 | 8.5 | -7.2 | -58 | 4 | -10 |
| 122 | -8.6 | 28.6 | 39.6 | -16 | 24 | 40 |
| 116 | -53.4 | 57.6 | 14.6 | -56 | 60 | 12 |
| 112 | 8.9 | 77.6 | -9.5 | 8 | 80 | -12 |
| 99 | 0.8 | 12.5 | 9 | -2 | 14 | 10 |
| 95 | 42.1 | 60.9 | -43.8 | 42 | 60 | -44 |
| 94 | 27.3 | 74.2 | 33.6 | 30 | 80 | 32 |
| 93 | 53.6 | -6.6 | -9.1 | 52 | -8 | -6 |
| 91 | -57.3 | -5.7 | 6.6 | -62 | -8 | 6 |
| 86 | 49.1 | 27.7 | 19.3 | 48 | 24 | 18 |
| 83 | 29.1 | -50 | 30 | 38 | -42 | 34 |
| 82 | 64.4 | 32.9 | -7.2 | 66 | 32 | -4 |
| 79 | -59.4 | 40.1 | 2.8 | -60 | 40 | 4 |
| 75 | -24.6 | -2.4 | -12.4 | -24 | -4 | -12 |
| 71 | 43.3 | 56.3 | 28.9 | 38 | 56 | 26 |
| 69 | -46.8 | 2.6 | 52.5 | -46 | 8 | 54 |
| 63 | 26.6 | 47.4 | -15.2 | 24 | 48 | -16 |
| 63 | -15.9 | 52.1 | 64.8 | -14 | 52 | 66 |
| 62 | 24.1 | -42.4 | 41.6 | 22 | -44 | 44 |
| 62 | 10.8 | 70 | 58.4 | 6 | 68 | 58 |
| 62 | 8.9 | 58.9 | 66.6 | 10 | 58 | 70 |
| 59 | 49.5 | -12.8 | 0.3 | 48 | -16 | -2 |
| 56 | 11.7 | 67.3 | -44.1 | 12 | 66 | -46 |
| 56 | 36.2 | -51.5 | 15.5 | 36 | -54 | 14 |
| 55 | 27 | 1.2 | -10.2 | 26 | -2 | -14 |

**Supplementary table 3: Face statistics.** Detailed statistics for each face, indicating the number of times each face was voted as good or bad by the viewers of the two video types separately, and the overall statistics. *The faces showing significant bias (1-sided binominal test ; p-value <0.05) to be chosen as good

| face indx | # of votes as good (video 1) | # of votes as bad (video1) | Sum  (video1) | # of votes as good (video2) | # of votes as bad (video2) | Sum  (video2) | Good  #votes | Bad  #votes | Good (percent) | Bad  (percent) | p-value  1-sided binominal test |
| --- | --- | --- | --- | --- | --- | --- | --- | --- | --- | --- | --- |
| 2 | 2 | -14 | 16 | 14 | -1 | 15 | 16 | 15 | 0.529 | 0.4708 | ns |
| 5 | 3 | -14 | 17 | 16 | 0 | 16 | 19 | 14 | 0.588 | 0.412 | ns |
| 7 | 2 | -15 | 17 | 15 | -1 | 16 | 17 | 16 | 0.528 | 0.472 | ns |
| 9 | 6 | -10 | 16 | 11 | -5 | 16 | 17 | 15 | 0.531 | 0.469 | ns |
| 14 | 13 | -4 | 17 | 10 | -7 | 17 | 23 | 11 | 0.676 | 0.324 | 0.03* |
| 24 | 4 | -12 | 16 | 13 | -3 | 16 | 17 | 15 | 0.531 | 0.469 | ns |
| 25 | 13 | -4 | 17 | 2 | -14 | 16 | 15 | 18 | 0.445 | 0.555 | ns |
| 28 | 6 | -11 | 17 | 15 | -2 | 17 | 21 | 13 | 0.618 | 0.382 | ns |
| 30 | 12 | -5 | 17 | 5 | -10 | 15 | 17 | 15 | 0.520 | 0.480 | ns |
| 32 | 4 | -13 | 17 | 16 | 0 | 16 | 20 | 13 | 0.618 | 0.382 | ns |
| 35 | 15 | -1 | 16 | 2 | -14 | 16 | 17 | 15 | 0.531 | 0.469 | ns |
| 36 | 4 | -13 | 17 | 11 | -4 | 15 | 15 | 17 | 0.484 | 0.516 | ns |
| 37 | 1 | -16 | 17 | 13 | -3 | 16 | 14 | 19 | 0.436 | 0.564 | ns |
| 38 | 4 | -12 | 16 | 14 | -2 | 16 | 18 | 14 | 0.562 | 0.438 | ns |
| 40 | 6 | -10 | 16 | 16 | -1 | 17 | 22 | 11 | 0.658 | 0.342 | 0.04* |
| 42 | 14 | -3 | 17 | 5 | -11 | 16 | 19 | 14 | 0.568 | 0.432 | ns |
| 45 | 16 | -1 | 17 | 3 | -14 | 17 | 19 | 15 | 0.559 | 0.441 | ns |
| 47 | 16 | -1 | 17 | 5 | -11 | 16 | 21 | 12 | 0.627 | 0.373 | ns |
| 49 | 4 | -13 | 17 | 13 | -4 | 17 | 17 | 17 | 0.5 | 0.5 | ns |
| 52 | 16 | -1 | 17 | 4 | -12 | 16 | 20 | 13 | 0.596 | 0.404 | ns |
| 55 | 12 | -4 | 16 | 8 | -9 | 17 | 20 | 13 | 0.610 | 0.390 | ns |
| 57 | 12 | -5 | 17 | 5 | -10 | 15 | 17 | 15 | 0.520 | 0.480 | ns |
| 59 | 13 | -4 | 17 | 4 | -12 | 16 | 17 | 16 | 0.507 | 0.493 | ns |
| 62 | 16 | -1 | 17 | 1 | -16 | 17 | 17 | 17 | 0.5 | 0.5 | ns |

**Supplementary table 4: Overlap of activation maps and ROIs.** The percentages of coverage of face-processing areas by other face-processing ROIs or by the resting-state functional connectivity map for the CDt

| Map1 | Map2 | A(Map1) $\cap$ A(Map2)* / A(Map2)  Right hemisphere | A(Map1) $\cap$ A(Map2) / A(Map2)  left hemisphere |
| --- | --- | --- | --- |
| Face localizer | Atlas FFA | %85.4 | %41.3 |
| CDt connectivity | Atlas FFA | %35.9 | %9.3 |
| CDt connectivity | Face localizer | %25.1 | %5.2 |

*A(Map1), A(Map2): area of each map

*A(Map1) $\cap$ A(Map2) : Intersection of areas for each map
